# Pioneer tree species accelerate restoration of tree‐related microhabitats in 50‐year‐old reserves of Białowieża Forest, Poland

**DOI:** 10.1002/ece3.10238

**Published:** 2023-07-03

**Authors:** Andreea Petronela Spînu, Weronika Mysiak, Jürgen Bauhus, Kamil Bielak, Mats Niklasson

**Affiliations:** ^1^ Faculty of Environment and Natural Resources University of Freiburg Freiburg Germany; ^2^ Southern Swedish Forest Research Centre Swedish University of Agricultural Sciences Alnarp Sweden; ^3^ Department of Silviculture Warsaw University of Life Sciences Warsaw Poland

**Keywords:** biodiversity conservation, biodiversity indicator, closer‐to‐nature forest management, compartmentalization of decay, forest restoration, pioneer species

## Abstract

Retention of structural elements such as deadwood and habitat trees at the level of forest stands has been promoted to integrate biodiversity conservation into multiple‐use forest management. The conservation value of habitat trees is largely determined by the presence, richness, and abundance of tree‐related microhabitats (TreMs). Since TreMs are often lacking in intensively managed forests, an important question of forest conservation is how the abundance and richness of TreMs may be effectively restored. Here, we investigated whether the strict protection of forest through cessation of timber harvesting influenced TreM occurrence at tree and stand levels. For that purpose, we compared four managed and four set‐aside stands (0.25 ha each) in the Białowieża Forest, with identical origin following clear‐cuts approximately 100 years ago. We found that the abundance and richness of TreMs on living trees were not significantly different between stands that were either conventionally managed or where active forest management ceased 52 years ago. Yet, our analysis of TreMs on tree species with contrasting life‐history traits revealed that short‐lived, fast‐growing species (pioneers) developed TreMs quicker than longer‐lived, slower‐growing species. Hence, tree species such as *Populus* or *Betula*, which supply abundant and diverse TreMs, can play an important role in accelerating habitat restoration.

## Graphical abstract

Tree‐related microhabitats (TreMs) are used as proxies for forest biodiversity levels and as practical tools for habitat tree selection. The necessary time for TreMs to develop though and consequently for forest biodiversity levels to recover is still relatively unknown. Our analysis captured the slow development of habitat structures in a 100‐year‐old forest reserve, where management ceased 52 years ago. Our findings underline the importance of pioneer species to accelerate restoration processes by providing abundant and rich TreMs quicker than long‐lived tree species. Photo credit: João Pereira.

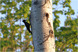

## 1 INTRODUCTION

Alternative forest management strategies have been discussed and implemented to maintain or restore forest biodiversity worldwide (FAO Report, 2020; Muys et al., 2022). To reduce the negative consequences of forest harvesting on habitats, management practices have been seeking to mimic the structures and biological legacies of natural disturbances, essential to ensure habitat continuity for many species (Franklin et al., 2002; Gustafsson et al., 2012). Whether the practices follow a segregative (i.e., land sparing—Paul & Knoke, 2015) or integrative paradigm (i.e., land sharing—Kraus & Krumm, 2013), a common denominator is the retention of specific areas and structural elements left in managed forests to develop into habitats for forest‐dwelling species. Biodiversity is scale‐dependent from a spatio‐temporal point of view and varies from micro‐to continental and daily to epochal scales (Brumelis et al., 2011). At the stand scale, retention of downed deadwood and habitat trees (large, old living, or dead trees that bear microhabitats) can be integrated in a wide range of different forest management approaches ranging from clearfelling to continuous‐cover systems (Gustafsson et al., 2012, 2020; Muys et al., 2022). At the landscape scale, forest areas are set‐aside as reserves, with either the goal of species protection, habitat restoration or preservation of natural forest dynamics (Angelstam et al., 2020; Muys et al., 2022). Small differences in the amount or the attributes of deadwood and habitat trees can affect the quality of reserves for specialized species (Bouget et al., 2014; Fritz & Heilmann‐Clausen, 2010). However, the necessary time for old‐growth structures to develop (e.g., habitat trees with particular tree‐related microhabitats—TreMs) and consequently for forest biodiversity to recover is case‐dependent (Vandekerkhove et al., 2011). The success to restore structural elements and communities of forest‐dwelling species reliant on those structures, depends on the starting conditions (e.g., the degree of naturalness, connectivity), the approach employed (passive or active), and the species colonization ability (Boeraeve et al., 2018; Herrault et al., 2016; Sabatini et al., 2020; Vandekerkhove et al., 2011). Provided that habitat requirements are met, the recolonization is prompt for species with high dispersal ability (e.g., birds, fungi; Vandekerkhove et al., 2011) but can be long‐delayed for slow‐colonizing species (e.g., vascular plants; Jackson & Sax, 2010).

Furthermore, in temperate Europe, forest conservation efforts and related research are often focused on the enhancement of old‐growth structures in mature stands, mostly dominated by long‐lived tree species such as *Fagus* or *Quercus*. Studies investigating the effect of management cessation on habitat provisioning highlighted the slow recovery of many forest‐dwelling species (e.g., saproxylic invertebrates) in such stands, often taking more than 100 years (Larrieu et al., 2017, 2019; Paillet, Pernot, et al., 2015; Vandekerkhove et al., 2005). Thus, in these cases or in forests with impoverished biodiversity (e.g., young even‐aged stands), a passive restoration approach is insufficient since it could take centuries to provide desired results (Sabatini et al., 2020). Consequently, active restoration efforts could be employed to accelerate habitat development. For example, tree “veteranisation” could speed up tree‐related microhabitat formation (Bengtsson et al., 2012; Menkis et al., 2022).

The presence, abundance and richness of TreMs, which are crucial for the life cycles of many forest‐dwelling species in terms of breeding, foraging, nesting, or hiding spaces, have been used to guide selection of valuable habitat trees during silvicultural operations in European forests (Asbeck et al., 2021; Larrieu et al., 2018). In addition, such tree‐ and stand‐level attributes of TreMs have been used as indicators of naturalness and to assess the effectiveness of retention forestry or restoration projects not only in Europe but also in North America (Asbeck et al., 2021; Larrieu et al., 2017; Martin et al., 2021, 2022). Many studies have shown that the main tree‐level drivers of TreM abundance and richness were tree species, diameter at breast height (DBH) and status (live or dead), along with interactions of these factors (e.g., Asbeck et al., 2019; Kaufmann et al., 2018; Michel & Winter, 2009; Paillet et al., 2017; Spînu et al., 2022; Vuidot et al., 2011). Environmental conditions (forest type, altitude) or management decisions are also linked to the TreM abundance and richness, which are by and large highest in unmanaged stands and primary forests (Asbeck et al., 2022; Larrieu et al., 2014b, 2014a). Commonly, large, old, and/or dead trees provide the most diverse and abundant TreMs, with often highest values for broadleaf species. Few studies tried to disentangle whether the positive effect of large tree dimension is directly related to a larger surface area or to its covariation with tree age and senescence (Kõrkjas et al., 2021a; Kozák et al., 2023; Puverel et al., 2019; Ranius et al., 2009). Tree senescence varies among tree species and is likely associated with the capacity of a tree to resist to or heal injuries. Since numerous TreMs are wounds, defects and often, structures related to wood decay, their formation and development relate to the tree capacity to compartmentalize injured tissues (described by Shigo & Marx, 1977). The compartmentalization of decay is associated with the chemical and physical wood properties (Shigo & Marx, 1977; Smith, 2015). For example, the presence of heartwood (e.g., in *Quercus* or *Pinus* species) or resin ducts (in Pinaceae) increases the resistance to microbial decay, protects the wood from pathogens, and aids quick wound healing (Smith, 2015). Diffuse‐porous tree species (e.g., *Acer*, *Betula*) do not produce heartwood and are less decay‐resistant than ring‐porous broadleaved species (e.g., *Quercus*, *Fraxinus*, or *Castanea*; Smith, 2015). Thus, forms of TreMs related to wood decay (e.g., mold cavities) are more likely to occur on diffuse‐porous tree species. In addition, the capacity to compartmentalize decay in wood decreases with the developmental stage of the tree (Smith, 2015). Fast‐growing pioneer species (e.g., *Betula*, *Populus*) reach mature and senescent ontogenic stages earlier than slower‐growing species and can develop TreMs that emerge from wounds or breakage already by the age of 100 years. In long‐lived, slower‐growing species such as *Quercus robur* L. it might take several hundred years for senescence processes to occur and TreMs to develop. Models of TreM formation based on more than 80,000 living trees also underlined the distinct TreM profile of early‐successional tree species such as *Populus* or *Betula* (high formation rates of bark loss, burr canker, crown deadwood, root concavity, and rot‐holes; Courbaud et al., 2022). Thus, restoring tree‐related microhabitats might be faster with pioneer tree species than with slower‐growing tree species. Pioneers may play that role in young forests or disturbed patches in a matrix of older forest (Przepióra & Ciach, 2022).

In this study, we investigated the temporal development of TreMs on coexisting trees with contrasting life‐history traits, in particular their successional character and wound compartmentalization capacity, as influenced by the cessation of active forest management. We compared TreM abundance and richness on living trees in Białowieża Forest, Poland, in four unmanaged secondary forest stands located in the Władysław Szafer's Forest Landscape Reserve (established in 1969), and four managed adjacent comparable stands with the same origin but under active management up to now. All stands represent nearly 100‐year‐old second‐growth forests that regenerated naturally in areas that were clearfelled between 1924 and 1929. Specifically, we addressed the following hypotheses:

- Owing to the repeated removal of trees, preferably those with defects, through thinning, living trees in stands where active forest management was terminated 52 years ago, provide more and richer TreMs than in nearby conventionally managed stands of the same age.
- The abundance and richness of TreMs are influenced by tree species life‐history traits, such as the successional character and wound compartmentalization capacity: pioneer species with low compartmentalization capacity provide rich, diverse TreM assemblages more quickly than long‐lived, slower‐growing species with higher compartmentalization capacity, irrespective of forest management.

The study thus allowed us to examine whether and how the cessation of forest management, which is increasingly adopted as an approach to biodiversity conservation, supports the restoration of tree‐related microhabitats. This information would be helpful to support restoration strategies for TreMs based on life‐history traits of trees.

## 2 METHODS

### 2.1 Research area

**FIGURE 1:**
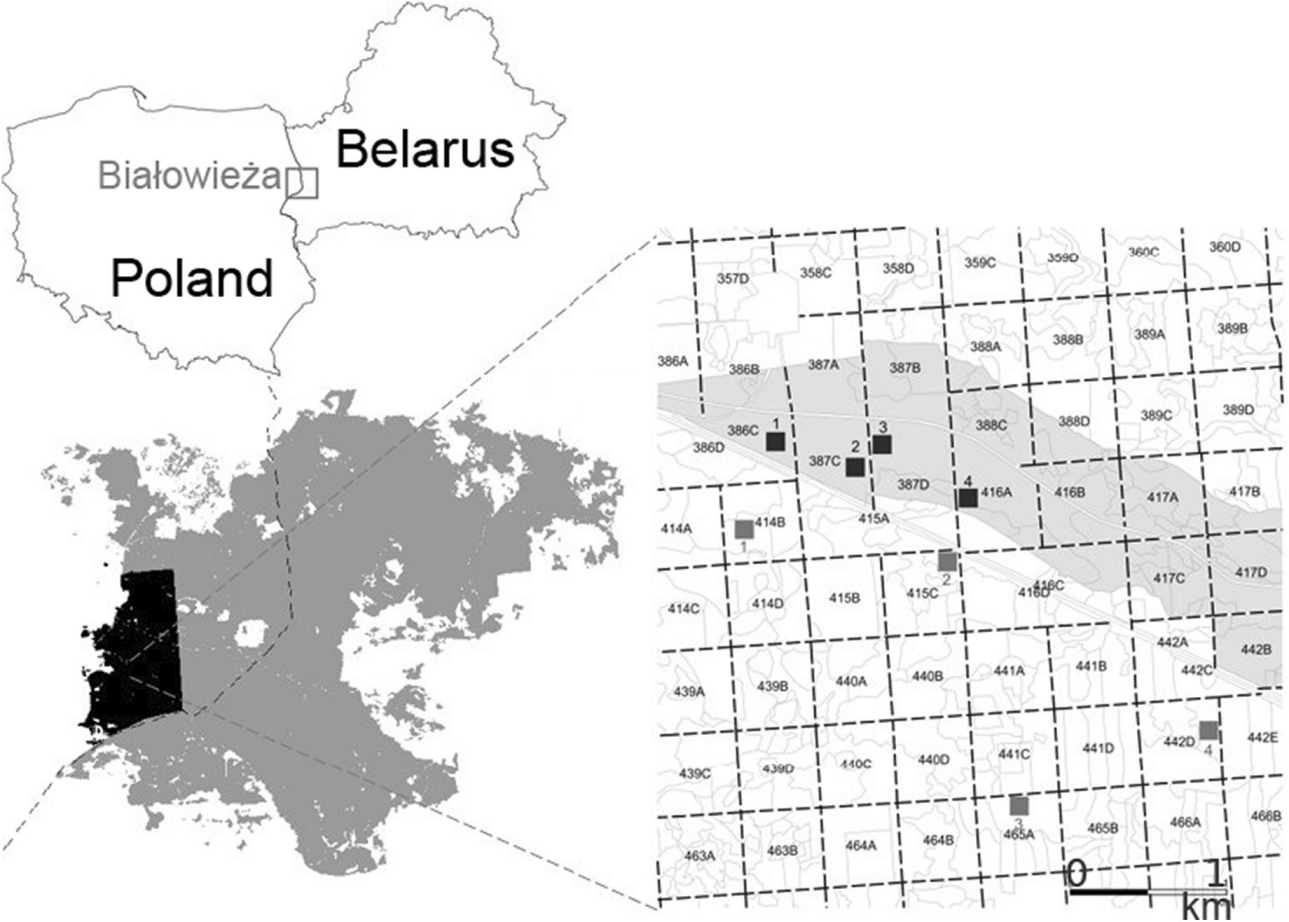
Location of the Białowieża Forest (Poland), study area and the eight research plots. Four of these plots (black squares) were in the unmanaged forests of the Władysław Szafer's Landscape Reserve (gray color, right panel) and another four in the managed part of Białowieża Forest, indicated by gray squares. Map data source: Forest Data Bank, Poland, https://www.bdl.lasy.gov.pl/portal/en.

This study was conducted in the Białowieża Forest in Poland, located near the border with Belarus (Figure 1). Approximately 50% of Białowieża Forest is strictly or partially protected and consists of old‐growth forest stands with longer or shorter history without active forest management (Krzyściak‐Kosińska et al., 2012; Sokołowski, 2004). Even though this area is now recognized as one of the best‐preserved lowland temperate forests in Europe, parts of it were clear‐cut by German occupation authorities during the First World War and later by the British company “Century” between 1924 and 1929 (Bielak, 2010; Mysiak, 2021). Subsequently, most areas regenerated naturally by fast‐growing, shade‐intolerant tree species like *Populus tremula* L., *Betula pendula* L., *Alnus glutinosa* (L.) Gaertn. With canopy closure, shade‐tolerant tree species appeared, dominating the regeneration up to the present (*Picea abies* (L.), Karst, *Quercus robur* L., *Acer platanoides* L., *Tilia cordata* Mill., and *Carpinus betulus* L.; Borecki & Brzeziecki, 2001; Paluch & Bielak, 2009). Such second‐growth forest stands are under‐studied (Borecki & Brzeziecki, 2001) and represent a unique opportunity to investigate the structural and functional differences of coexisting tree species, with contrasting life‐history traits.

We selected eight 0.25 ha plots in second‐growth forests with similar site conditions (brunic arenosol soil type formed on a loamy sand). The plots are part of a network of 50 plots under observation for the last 20 years by the Department of Silviculture, Warsaw University of Life Sciences in Poland (Bielak, 2010). Four plots are located in unmanaged stands of Władysław Szafer's Landscape Reserve, where active forest management ceased in 1969. The other four plots are located in nearby managed stands (Figure 1; Appendix S1). Since the end of the Second World War, regular silvicultural treatments were carried out in the managed areas (i.e., first cleanings up to 20 years old followed by later thinnings). Over the last 80 years, regular crop thinnings were carried out favoring more economic valuable long‐lived species (*Q. robur*, *P. abies*, *T. cordata* and *A. platanoides*) over less desirable mature and over‐mature pioneer tree species like *P. tremula* and *B. pendula* (Bielak & Brzeziecki, 2006). However, in stands dominated by pioneer tree species and *C. betulus*, where desired crop trees were scarce (<100–150 trees ha^−1^), the practices aimed to reduce competition between pioneer tree species. Thinnings were repeated regularly (every 10 years), with a low‐to‐moderate intensity (up to 10%–15% of total merchantable volume over bark).

The mean density of all living trees (DBH ≥ 5 cm) was 627 (SE ± 53) trees ha^−1^ in managed plots and 798 (±92) trees ha^−1^ in the case of unmanaged plots. The mean basal area was 33.2 (±2.3) m^2^ ha^−1^ and 34.9 (±2.1) m^2^ ha^−1^ in managed and unmanaged plots, respectively. The stocking density was higher in the stands that were unthinned over the last 50 years and consequently, had a lower mean DBH in comparison with the managed stands (23.5–26.0 cm). The density and basal area of the standing dead trees differed as well between the two stand categories with four to five times higher values in the unmanaged plots: 120 dead trees ha^−1^ and 1.8 m^2^ ha^−1^ in the unmanaged plots in contrast to 23 dead trees ha^−1^ and 0.4 m^2^ ha^−1^ in managed stands (Bielak, 2010, inventory data updated in 2020).

The study area is dominated by trees species with different life‐history traits such as life expectancy, successional character, and wound compartmentalizing capacity, which can have a significant effect on TreM formation. According to tree‐ring data, *Q. robur* in Białowieża Forest reaches 400–450 years whereas *P. tremula* and *B. pendula* can live up to 200–250 years. While the maximum lifespan for *T. cordata* is unknown due to the lack of intact tree‐ring series (large trees are usually hollowed), *A. platanoides*, *P. abies* and *C. betulus* in the study area can become 300–350 years old (E. Zin, unpublished; Spînu et al., 2020). Furthermore, *P. tremula*, *B. pendula*, *A. platanoides*, *C. betulus* have a lower wound compartmentalization capacity and higher decay rate than *P. abies* and *Q. robur* due to their wood properties (Kahl et al., 2017; Smith, 2015). Accordingly, we classified the tree species in this study into three groups regarding their TreM development potential: short‐lived, fast‐growing pioneer species with a low compartmentalization capacity (*P. tremula*, *B. pendula*); intermediate compartmentalization capacity (*A. platanoides*, *T. cordata*, *C. betulus*, *P. abies*); and long‐lived, slow‐growing with a high compartmentalization capacity (*Q. robur*).

### 2.2 Data collection and analysis

In each study plot, a TreM survey was carried out on all living trees with a DBH >20 cm. Data were collected during a snow‐free period in spring 2021, when the leafless crowns of deciduous trees could best be visually surveyed for TreMs. These surveys were carried out by the same person to avoid observer bias (Paillet, Coutadeur, et al., 2015; Paillet, Pernot, et al., 2015). TreMs were recorded following the standardized typology of Larrieu et al. (2018), with 15 hierarchical groups and 47 types, relevant to forest‐dwelling species (Appendix S2). The summary of the main characteristics of surveyed trees are presented in Tables 1 and 2.

**TABLE 1:** Stand‐level characteristics of the inventoried trees (total number, basal area, and share of different tree species).

	No. trees ha^−1^	Basal area average ± SD (m^2^ ha^−1^)	Tree species share (%)						
			*Acer platanoides*	*Betula pendula*	*Carpinus betulus*	*Picea abies*	*Populus tremula*	*Quercus robur*	*Tilia cordata*
Managed	334	28.2 ± 4.4	0.3	28.1	38.9	13.2	1.5	12.0	6.0
Unmanaged	338	26.7 ± 4.5	0	43.5	26.9	16.0	0.6	12.7	0.3

**TABLE 2:** Main attributes of the inventoried trees based on stand management and species life‐history traits.

Tree species senescence classes based on life‐history traits	No. trees	Total share (%)	DBH (mm)				TreM abundance per tree			TreM richness per tree		
			Mean	SD	Min.	Max.	Mean	SD	Max.	Mean	SD	Max.
Managed (*N* = 334)												
Short‐lived, fast‐growing	99	29.6	38.9	10.1	21.2	70.7	3.4	7.2	45	0.8	0.9	5
Intermediate	195	58.4	27.1	6.8	20.0	59.7	0.9	1.8	18	0.7	0.9	5
Long‐lived, slow‐growing	40	12.0	32.9	7.9	20.0	49.2	1.5	2.9	17	0.9	1.1	5
Unmanaged (*N* = 338)												
Short‐lived, fast‐growing	149	44.1	35.7	8.1	20.0	57.4	4.5	7.5	38	1.1	1.1	7
Intermediate	146	43.2	26.4	6.2	20.0	50.4	0.8	1.2	6	0.8	0.8	3
Long‐lived, slow‐growing	43	12.7	26.8	6.6	20.0	48.6	0.8	0.7	3	0.8	0.7	2

Data analysis was performed using R 1.4.1717 (R Core Team, 2021). A preliminary investigation of outliers, heterogeneity of variance, collinearity, and missing values was carried out to avoid type I and II errors according to the protocol of Zuur et al. (2010). Total abundance was defined as the number of individual TreMs per tree and total richness as the total number of different TreM groups per tree. To investigate whether forest management, DBH and tree species group were significant predictors of tree‐level TreM abundance and richness, generalized linear mixed models at the tree level with plot as random factor (GLMMs) were fitted using the R package “glmmTMB” (Brooks et al., 2017). A Poisson error distribution was suitable only for the *TreM richness* and the *Rot‐holes* models. For the rest of the models, we used negative binomial distributions. The under‐ and overdispersion as well as zero inflation in the models were tested with the “DHARMa package” (Hartig, 2018). Previous papers highlighted a strong site effect on TreM occurrence (Larrieu et al., 2022). In our study, we focused on the influence of management on TreMs in forests of similar tree species composition. While it would have been interesting to investigate the interactions between management and site factors, it would be very difficult to implement such a design in the field, which would need to be based on forests of the same age and similar species composition in both managed and unmanaged condition and across a variety of site types. However, since there were no obvious site differences among our study plots in terms of soil type or topography, we did not associate a hypothesis with site effects. However, to account for possible differences related to the influence of site conditions, the plot (site identity) was included as a random effect in the model.

Seven out of fifteen TreM groups were represented by a sufficient number of observations for each GLMM (>20 observations): rot‐holes, concavities, exposed sapwood, exposed sap‐ and heartwood, crown deadwood, epiphytes, and microsoils. The other TreM groups were considered for calculating tree‐level TreM abundance and richness. The final nine models (*~DBH + Forest Management + Tree species class + (1|PlotID*)) are summarized in Table 2 and outlined in detail in Appendix S3. Model predictions based on the significant predictors and the color‐blind friendly visualizations were performed with the “ggeffects,” “ggplot,” “viridis” R packages (Garnier et al., 2021; Lüdecke, 2018; Wickham, 2016). At the stand level, we tested whether values of total TreM abundance and richness were significantly higher in unmanaged forests than those in managed forests by employing nonparametric methods (Mann–Whitney–Wilcoxon), since the data did not comply with the requirements of parametric methods. We used an alpha level of .05 for all statistical tests.

## 3 RESULTS

**FIGURE 2:**
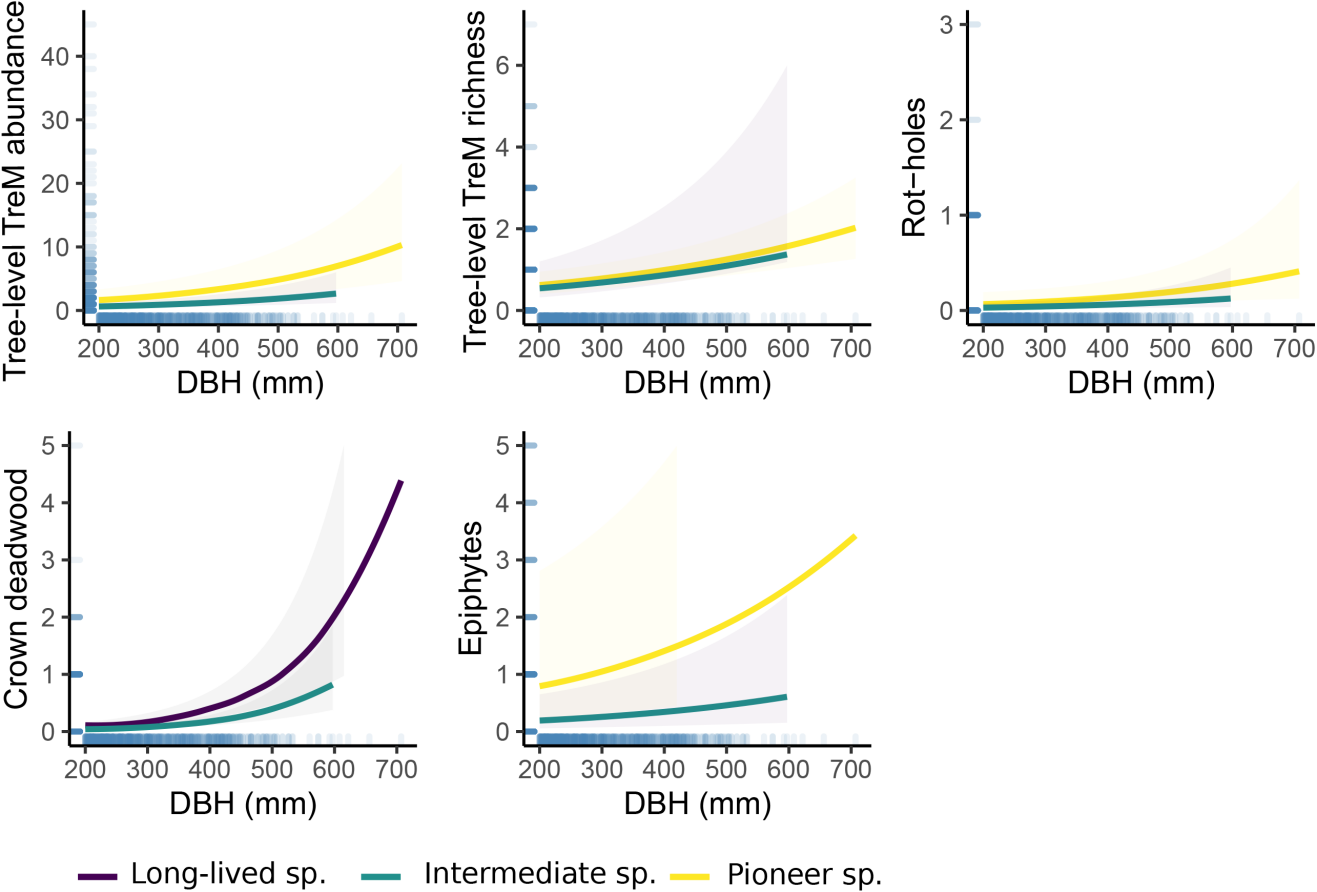
Tree‐related microhabitat (TreM) estimates (tree‐level abundance and richness, counts for each TreM group) of surveyed trees in response to tree diameter at breast height and tree species group (based on successional character and compartmentalization capacity). Ribbons represent the 95% confidence intervals. Only significant results are included in the graphs. Forest management was not a significant predictor in any of the models.

**FIGURE 3:**
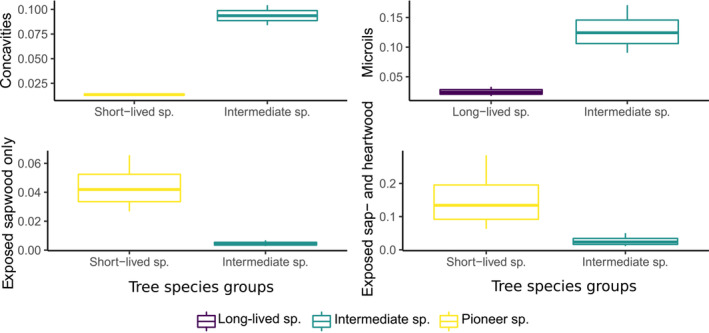
Estimated effect of tree species groups (based on successional character and compartmentalization capacity) on mean tree‐related microhabitat group abundance, at tree level. Tree diameter at breast height and forest management were not significant predictors. Only significant results are included in the graphs.

At the tree level, forest management was not a significant predictor of TreM abundance or richness in any of the GLMMs (Table 3). Tree species groups were important predictors for the tree‐level TreM richness, abundance and most TreM groups, together with tree dimension (Table 3; Figures 2 and 3). Large individuals of pioneer species with low compartmentalization capacity (*Populus*, *Betula*) were associated with the highest TreM abundance and richness. We additionally tested for the effect of interaction between tree dimension and species class on tree‐level TreM abundance and richness. This analysis revealed that with increasing DBH, TreM richness increased more strongly on trees of long‐lived species than on pioneers, whereas TreM abundance remains mostly driven by the pioneer species. Large individuals of species such as *Populus* and *Betula* were associated as well with the occurrence of some TreM groups, for instance of rot‐holes and epiphytes. While DBH is an important driver of TreM occurrence in most studies (Asbeck et al., 2021; Courbaud et al., 2022; Larrieu et al., 2022; Martin et al., 2022), it was not a significant predictor for concavities, exposed sapwood and exposed sap‐ and heartwood occurrence in this study. The two latter TreM groups were found on pioneer tree species, similar to the overall TreM abundance and richness. The highest abundance of concavities and microsoils though, was predicted for the intermediate tree species group (*Acer*, *Tilia*, *Carpinus*, *Picea*; Figure 3). Long‐lived, slow‐growing species with a high compartmentalization capacity (*Quercus*) supported more crown deadwood than other species (Figure 2).

**TABLE 3:** Results of the final generalized linear mixed models indicating the magnitude of influence and the significance* of the predictors DBH, forest management, and tree species classes (long‐lived, slow‐growing/ intermediate /short‐lived, fast‐growing sp.).

	Intercept	DBH (cm)	Unmanaged stand	Tree category	
				Long‐lived, slow‐growing sp.	Short‐lived, fast‐growing sp.
Tree‐level TreM abundance^a^	−1.21*	0.01*	−0.03	−0.08	0.52*
Tree‐level TreM richness^b^	−1.07*	0.01*	0.08	−0.02	0.113
Rot‐holes^b^	−4.25*	0.01*	−0.57	0.27	0.79*
Concavities^a^	−2.56*	0.01	0.17	−20.46	−1.94*
Exposed sapwood^a^	−4.63*	−0.01	0.71	0.22	2.26*
Exposed sap‐ and heartwood^a^	−2.39*	−0.01	−0.90	−1.08	1.73*
Crown deadwood^a^	−4.89*	0.01*	0.16	0.82*	−0.01
Epiphytes^a^	−2.23*	0.01*	0.45	0.19	1.41*
Microsoils^a^	−2.65*	0.01	−0.23	−1.63*	−0.39

*Note*: Positive values show an increase in the group of tree‐related microhabitats. The intercept represents the variables *Intermediate* sp. and *Managed stand*. A detailed description of all fitted models can be found in Appendix S3. Models fitted with negative binomial distributions (a) or Poisson distributions (b).

**FIGURE 4:**
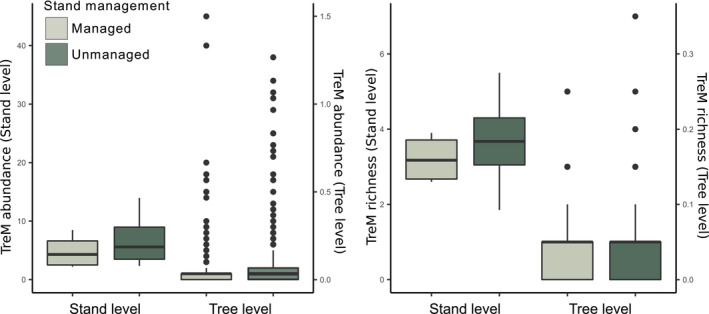
Estimated stand‐ and tree‐level mean of tree‐related microhabitat abundance (left) and richness (right) by stand management (95% confidence interval).

At the stand level, TreM abundance and richness did not differ significantly between the two forest management regimes (Mann–Whitney–Wilcoxon test) for TreM abundance (*W* = 6, *p*‐value = .67) and for TreM richness (*W* = 6,5, *p*‐value = .77; Figure 4).

## 4 DISCUSSION

Our findings show that living trees in forest stands that were unmanaged for five decades did not provide more and richer TreMs than those in managed stands of the same origin. TreM abundance and richness at the tree level were mainly driven by the successional character and wound compartmentalization capacity of tree species, irrespective of the forest management. Pioneer species with low compartmentalization capacity provided more and richer TreMs than longer‐lived, slow‐growing tree species with high compartmentalization capacity. Our results indicated that in the current stand development stage, long‐lived and pioneer trees have similar tree‐level TreM richness. However, the interaction between tree diameter and species class shows that long‐lived species accumulate more diverse TreMs than pioneer species for the same tree dimension. Owing to the small number of large trees, this result should be interpreted with caution, but it suggests that long‐lived tree species can maintain a diverse TreM composition once the pioneer species disappear from the stand in the course of succession (Palik et al., 2020). The combination of short‐lived, fast‐growing and long‐lived, slow‐growing species thus ensures a temporal complementary of TreM richness.

At the stand level, TreM estimates did not differ between the two management regimes. The latter result is not surprising, since tree species composition and tree dimensions were not significantly different between actively managed and unmanaged stands.

Our analysis indicates that a period of approximately 50 years since cessation of active forest management is not long enough to influence either the stand‐ or tree‐level development of TreMs (congruently to Asbeck et al., 2019; Vuidot et al., 2011). In contrast, differences between TreM richness and abundance are usually significant between managed and long‐term unmanaged forests (e.g., primary forests or old reserves; Asbeck et al., 2022). However, since management in our study ceased only 50 years ago, we assume that the similarity between the two treatment types may have been caused by early stand tending practices that were carried out before the treatments (cessation vs. continuation of management) were implemented. In young broadleaved stands, a common practice of negative selection is the removal of dominant trees of low quality (e.g., wolf trees) in a pre‐commercial tending operation before the selection of crop trees is possible. These are likely the trees with the highest TreM development potential (forked, twisted, branchy, injured, or diseased), which would have otherwise contributed to TreM abundance and richness in the unmanaged stands. In addition, the similarity between treatments may also be attributable to a change toward close‐to‐nature silvicultural principles in the Białowieża Forest in 1995. According to this management approach (e.g., Bauhus et al., 2013), thinning is based on a positive selection of crop trees that are promoted through the removal of the strongest competitors. This type of operation applied in the managed stands were of low to moderate intensity and (up to 10%–15% of total merchantable volume under bark). Thus, TreM‐bearing trees that were not competitors of crop trees would have been retained. However, trees of lower quality that have so far not been competitors of crop trees, may still be removed in future thinning interventions.

In our study, the occurrence of TreMs on living trees was to a large extent related to the tree species life‐history traits such as successional character and wound compartmentalization capacity. Interestingly, the highest tree‐level TreM abundance and richness was found on pioneer trees, which have the lowest compartmentalization capacity. At the TreM group level, rot‐holes, epiphytes, exposed sap‐ and heartwood were more likely to develop on *P. tremula* and *B. pendula*. This pattern indicates that even though these fast‐growing trees can live up to 200 years in the study area, they have reached a mature and senescent ontogenetic stage. For example, after 80 years, *P. tremula* supported richer TreMs than *P. abies* in Estonian forests (Kõrkjas et al.,  2021b). In short‐lived species, tree senescence likely begins at 100 years and larger branches start to break allowing fungi to penetrate and wood rot to form (Basham, 1991; Hunt & Etheridge, 1995). Since wood decay compartmentalization capacity decreases further with the ontogenetic stage (Smith, 2015), this process allows TreMs such as rot‐holes (e.g., trunk cavities with or without mold, hollow branches), exposed sapwood only (bark loss, shelters, pockets), and exposed sap‐ and heartwood (stem and limb breakage, cracks) to form. Also, in other studies, similar TreM groups were associated with early‐successional species, as *Populus* and *Betula* (bark loss, crown deadwood, and rot‐holes; Courbaud et al., 2022). In our study, crown deadwood was more likely to occur on long‐lived tree species. The probability of this TreM group to be abundant on species such as *Q. robur* is likely due to the high decay resistance of their wood, allowing dead branches to remain in the crown longer than in fast‐growing species, especially in old trees (Basham, 1991; Smith, 2015). Concavities, such as water‐filled holes (dendrotelmata) or root buttresses were predicted to occur more likely on intermediate tree species. In Central Europe, the majority of tree holes are found in *Fagus sylvatica* trees, followed by *Picea*, *Carpinus*, *Acer* and *Tilia* species (Gossner et al., 2015). These species, which represented in our study the intermediate tree species group (except for *F. sylvatica*, which was not present), are pivotal for the provisioning of concavities.

By sampling forest stands of the same age and origin, we were able to distinguish between the effect of DBH and tree age on TreM occurrence. So far, the positive effects of tree dimension on TreM estimates were understood as either an indirect effect of tree age or increased tree surface area (Asbeck et al., 2021). Few studies have sought to separate tree age and DBH effect on TreM development (Kõrkjas et al., 2021a ; Kozák et al., 2023). In congruence with numerous studies (Jahed et al., 2020; Paillet et al., 2019; Spînu et al., 2022), the tree‐level TreM abundance and richness increased with increased tree size and TreM groups associated with increased surface area (rot‐holes, crown deadwood, epiphytes) were positively affected by tree size. In contrast, tree DBH did not have a significant effect on the occurrence of concavities, exposed sap‐ and heartwood in our analysis. These TreM groups (e.g., dendrotelmata, woodpecker foraging excavations, stem, and fork level cracks) are more likely to be related to tree ontogeny and stem form rather than tree dimensions. For example, in primary forests, tree age had a greater effect than diameter for occurrence of exposed sapwood, insect galleries (in spruce forests), burrs, and cankers (in spruce and mixed‐beech forests; Kozák et al., 2023).

While pioneer tree species can provide TreMs quicker, they cannot replace the substrate‐specific TreMs in other tree species. Depending on the specific wood substrate (characterized by tree species, wood decay, sun exposure), TreMs such as crown deadwood and rot‐holes offer suitable resources for different taxa (tree species‐associated saproxylic wasps or beetles—Bouget et al., 2011; Ulyshen et al., 2011). Furthermore, endangered species such as the longhorn beetle (*Cerambyx cerdo*) rely on long‐lived oak trees for the survival of their populations, and this can impact other threatened species, for which the beetle shapes habitats (Brin & Bouget, 2018; Buse et al., 2008). The functionality of TreMs also relates to their rate of formation, development, and persistence. While many TreM types (e.g., cavities, exposed sap‐ and heartwood, crown deadwood) form more rapidly in pioneer species, they persist longer in slow‐growing trees (Kõrkjas et al.,  2021b). A TreM that persists in the long‐term could be utilized by many species during its development and contributes substantially to the pool of nesting and roosting resources (Edworthy et al., 2012). For example, a woodpecker cavity of 17 years has been used up to 20 times by six different bird species (Wesołowski & Martin, 2018). Thus, even though TreMs such as rot‐holes have a probability of occurrence of only 1% on 100‐year‐old oak trees, once formed on these slow‐growing trees at 200–300 years of age (Ranius et al., 2009), they can persist as suitable habitats for long periods, as long as the tree is standing. TreMs of pioneer tree species are likely to have shorter persistence rates because of early tree senescence and short lifespan. They can therefore disappear relatively quickly from stands in the process of forest succession, especially in the absence of stand‐opening disturbances (Palik et al., 2020). For instance, in the present conditions of many stands in the Białowieża Forest, it has been exclusively shade‐tolerant trees that shown successful establishment in the last decades (e.g., *Acer*, *Carpinus*; Brzeziecki et al., 2016; Kuijper et al., 2010; Spînu et al., 2020), which, as indicated by our analysis, do not provide as many and diverse TreMs as pioneer trees do. Consequently, the provision through pioneer species of TreMs for which the wood substrate may be less relevant (e.g., tree hollows important for vertebrates), requires a landscape perspective and must be part of a long‐term strategy to ensure habitat continuity.

In addition, one major driver of TreM occurrence is the tree status. For example, TreMs are often more abundant and diverse on dead trees than on living ones (Larrieu et al., 2012; Paillet et al., 2019) and their composition on dead trees can differ from that on living trees (Spînu et al., 2022). Thus, to complement our findings, which were focused on living trees only, future studies should investigate the effect of time since cessation of management on TreM development on both living and dead trees of different species and successional character.

## 5 CONCLUSION AND RECOMMENDATIONS

Our analysis indicated little differences in TreM occurrence between ca. 100 years old managed and unmanaged forests, where management ceased approximately 50 years ago. Early interventions and thinning procedures carried out before the reserve was established, may have removed potential habitat trees, and thus the present TreM abundance and richness may differ from forests that were unmanaged from the beginning. Thus, besides habitat tree retention in mature European forests for enhancing biodiversity (Muys et al., 2022), enhanced microhabitat provision may also be initiated at earlier stages of stand development. For example, during early thinning operations, potential habitat trees could then be recognized and already be retained (Kõrkjas et al.,  2021b) or TreMs could even be actively created (Bengtsson et al., 2012; Menkis et al., 2022). In addition, our findings underline the importance of pioneer tree species with low compartmentalization capacity to accelerate restoration processes by providing abundant and rich TreMs quicker than long‐lived, slow‐growing tree species. Our results also suggest that habitat trees should be selected from different tree species from different successional stages in order to achieve abundant and diverse TreMs at different time scales.

## AUTHOR CONTRIBUTIONS

**Andreea Petronela Spînu:** Conceptualization (lead); data curation (lead); formal analysis (lead); investigation (lead); methodology (lead); project administration (equal); software (lead); validation (lead); visualization (lead); writing – original draft (lead); writing – review and editing (lead). **Weronika Mysiak:** Data curation (supporting); formal analysis (supporting); investigation (supporting). **Jürgen Bauhus:** Conceptualization (equal); funding acquisition (equal); project administration (equal); supervision (equal); validation (equal); writing – original draft (supporting); writing – review and editing (supporting). **Kamil Bielak:** Conceptualization (equal); funding acquisition (equal); methodology (equal); project administration (equal); resources (equal); writing – original draft (supporting); writing – review and editing (supporting). **Mats Niklasson:** Conceptualization (equal); methodology (equal); project administration (equal); supervision (equal); validation (equal); writing – original draft (supporting); writing – review and editing (supporting).

## CONFLICT OF INTEREST STATEMENT

The authors declare there are no competing interests.

## Supporting information


Appendix S1

Data S1

## Data Availability

Data analyzed during this study are provided in full within the published article as Supporting Information.
