# Supplementary material for: Pioneer tree species accelerate restoration of tree‐related microhabitats in 50‐year‐old reserves of Białowieża Forest, Poland
Source: Ecol Evol. 2023 Jul 3;13(7):e10238. doi: 10.1002/ece3.10238 (PMC10316124; doi:10.1002/ece3.10238)

Appendix 1a. Study area – unmanaged stands.
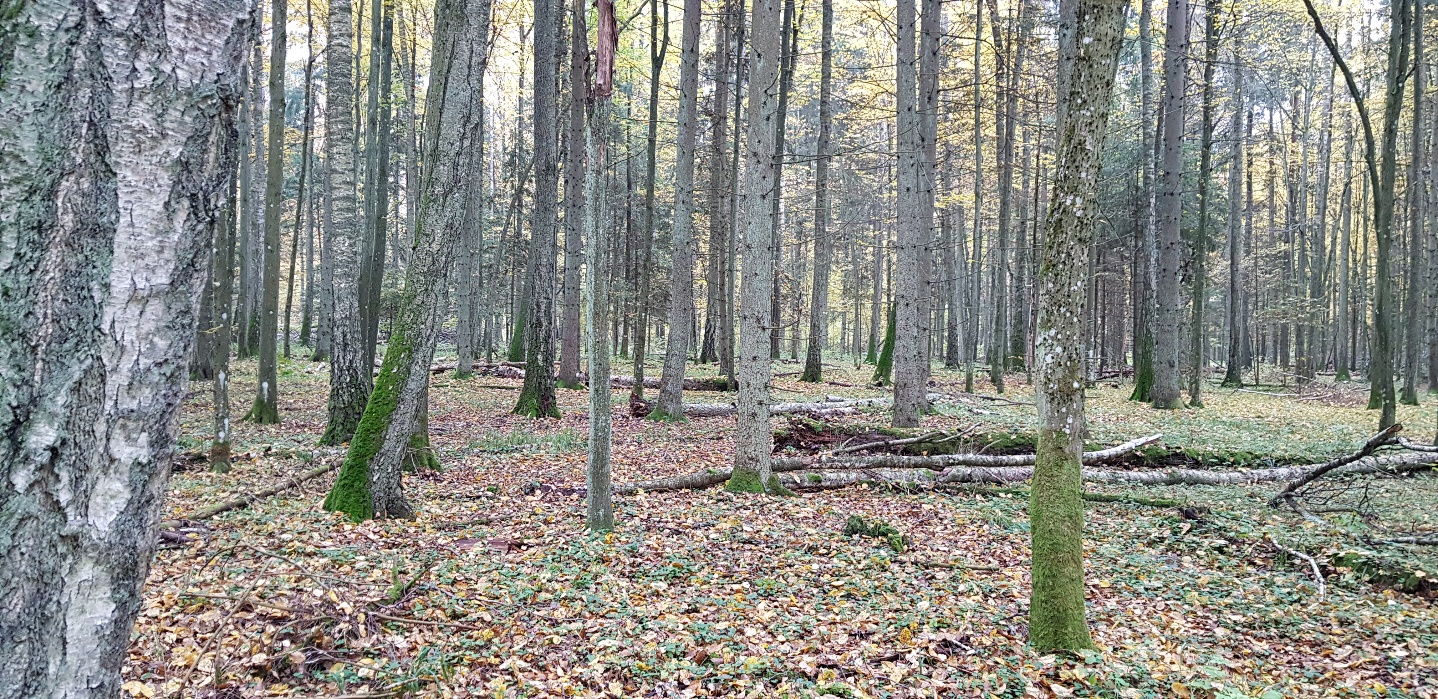


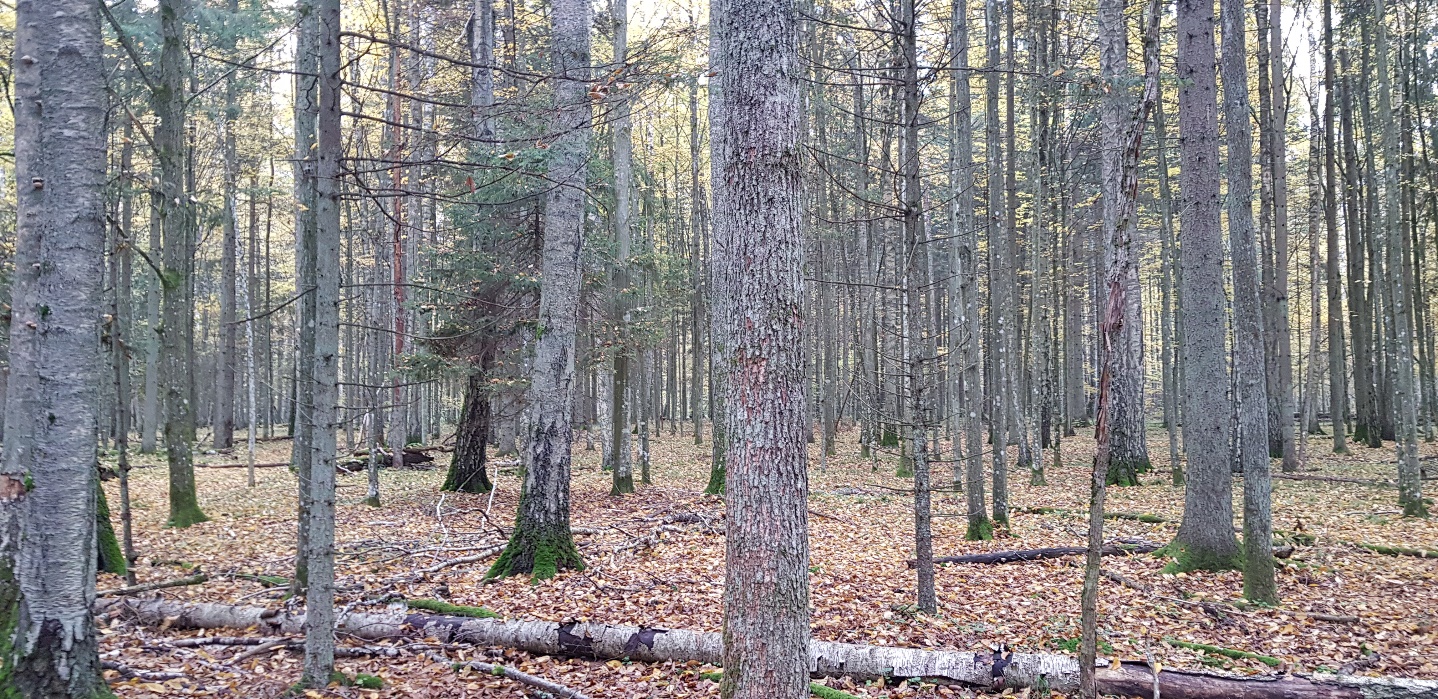


Appendix 1b. Study area – managed stand.
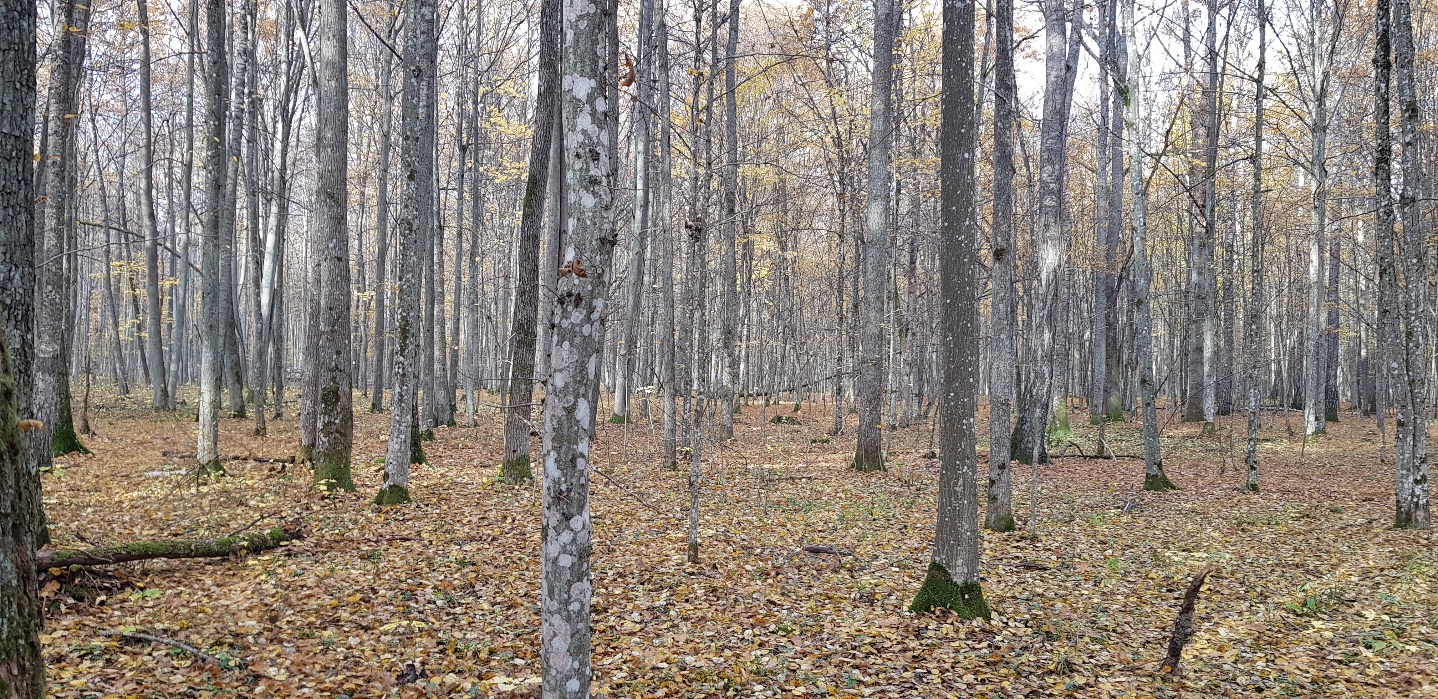


Appendix 2. Classification and description of tree-related microhabitats from Larrieu et al. 2018

| **Form** | **Group** | **Examples of TreM types** |
| --- | --- | --- |
| **Cavities** | Woodpecker cavities | Small woodpecker breeding cavity.  Medium-sized woodpecker breeding cavity  Large woodpecker breeding cavity  Woodpecker “flute” (breeding cavity string) |
|  | Rot-holes | Trunk base rot hole  Trunk rot hole  Semi-open trunk rot hole  Chimney trunk base rot hole  Chimney trunk rot hole  Hollow branch |
|  | Insect galleries and bore holes | Insect galleries and bore holes |
|  | Concavities | Dendrotelm (phytotelmata, waterfilled hole)  Woodpecker foraging excavation  Trunk bark-lined concavity  Root buttress concavity |
| **Tree injuries and exposed**  **wood** | Exposed sapwood only | Bark loss  Fire scar  Bark shelter  Bark pocket |
|  | Exposed sapwood and  heartwood | Stem breakage  Limb breakage (heartwood exposed)  Crack  Lightning scar  Fork split at the intersection |
| **Crown deadwood** | Crown deadwood | Dead branches  Dead top  Remaining broken limb |
| **Excrescences** | Twig tangles | Witch broom  Epicormic shoots |
|  | Burrs and cankers | Burrs and cankers |
| **Fruiting bodies of**  **saproxylic fungi and**  **slime moulds** | Perennial fungal fruiting bodies (life span> 1y) | Perennial polypore |
|  | Ephemeral fungal fruiting bodies and slime moulds | Annual polypore  Pulpy agaric  Pyrenomycete  Myxomycete |
| **Epiphytic, epixylic and**  **parasitic structures** | Epiphytic or parasitic crypto- and phanerogams | Bryophytes  Foliose and fruticose lichens  Ivy and lianas  Ferns  Mistletoe |
|  | Nests | Vertebrate nest  Invertebrate nest |
|  | Microsoils | Bark microsoil  Crown microsoil |
| **Fresh exudates** | Fresh exudates | Sap run  Heavy resinosis |
| **7 forms** | 15 groups | 47 types |

Appendix 3. Results of the final generalized linear mixed models, at tree-level.

| Model | | SE | *Z* value | Pr (>\|*z*\|) | Significance  *<0.05 | Model summary | | | | | | Final model performance by checking residuals and diagnostics, over-dispersion and zero-inflation with the ‘DHARMa’ package (Hartig, 2018) | | |
| --- | --- | --- | --- | --- | --- | --- | --- | --- | --- | --- | --- | --- | --- | --- |
| Variable | Estimate |  |  |  |  | Distribution | Conditional pseudo R squared (‘MuMIn’ package, Bartoń 2023) | AIC Final model /  AIC  Null model | logLik | deviance | Df.resid | Outliers test,  p value | Dispersion test,  p value | Zero-inflation test,  p value |
| **TreM abundance model** | | | | | | Negative binomial | 0.33 | 2239.0/2378 | -1112.5 | 2225.0 | 665 | 0.7 | 0.696 | 0.656 |
| Intercept | -1.2112 | 0.3915 | -3.093 | 0.0019 | * |  |  |  |  |  |  |  |  |  |
| DBH (cm) | 0.0036 | 0.0001 | 4.793 | 1.64e-06 | * |  |  |  |  |  |  |  |  |  |
| Unmanaged stand | 0.0340 | 0.4600 | 0.074 | 0.9409 |  |  |  |  |  |  |  |  |  |  |
| Long-lived, slow-growing sp. | -0.0832 | 0.1960 | -0.424 | 0.6712 |  |  |  |  |  |  |  |  |  |  |
| Short-lived, fast-growing sp. | 0.9529 | 0.1563 | 6.095 | 1.10e-09 | * |  |  |  |  |  |  |  |  |  |
| **TreM abundance model with interaction DBH: Tree species class** | | | | | | Negative binomial | 0.35 | 2230.7/2378 | -1106.4 | 2212.7 | 663 | 0.78 | 0.72 | 0.584 |
| Intercept | 0.01 | 0.526 | 0.021 | 0.98 |  |  |  |  |  |  |  |  |  |  |
| DBH (cm) | -0.001 | 0.001 | -0.602 | 0.54 |  |  |  |  |  |  |  |  |  |  |
| Unmanaged stand | 0.02 | 0.463 | 0.055 | 0.95 |  |  |  |  |  |  |  |  |  |  |
| Long-lived, slow-growing sp. | -1.36 | 0.77 | -1.751 | 0.07 |  |  |  |  |  |  |  |  |  |  |
| Short-lived, fast-growing sp. | -0.93 | 0.55 | -1.681 | 0.09 |  |  |  |  |  |  |  |  |  |  |
| DBH: Long-lived, slow-growing sp. | 0.01 | 0.001 | 1.834 | 0.06 |  |  |  |  |  |  |  |  |  |  |
| DBH: Short-lived, fast-growing sp. | 0.06 | 0.01 | 3.530 | 0.001 | * |  |  |  |  |  |  |  |  |  |
|  | | | | | | | | | | | | | | |
| **TreM richness model** | | | | | | Poisson | 0.18 | 1555.8/1584 | -770.9 | 1541.8 | 665 | 0.56 | 0.656 | 0..592 |
| Intercept | -1.0798 | 0.237 0 | -4.556 | 5.22e-06 | * |  |  |  |  |  |  |  |  |  |
| DBH (cm) | 0.0023 | 0.0001 | 4.602 | 4.17e-06 | * |  |  |  |  |  |  |  |  |  |
| Unmanaged stand | 0.086 | 0.2537 | 0.339 | 0.735 |  |  |  |  |  |  |  |  |  |  |
| Long-lived, slow-growing sp. | -0.0029 | 0.1408 | -0.021 | 0.983 |  |  |  |  |  |  |  |  |  |  |
| Short-lived, fast-growing sp. | 0.1333 | 0.1111 | 1.197 | 0.231 |  |  |  |  |  |  |  |  |  |  |
| **TreM richness model with interaction DBH: Tree species class** | | | | | | Poisson | 0.19 | 2239.0/2378 | -1112.5 | 2225.0 | 665 | 0.58 | 0.504 | 0.56 |
| Intercept | -0.25 | 0.333 | -0.768 | 0.44 |  |  |  |  |  |  |  |  |  |  |
| DBH (cm) | -0.01 | 0.001 | -0.716 | 0.74 |  |  |  |  |  |  |  |  |  |  |
| Unmanaged stand | 0.114 | 0.251 | 0.455 | 0.64 |  |  |  |  |  |  |  |  |  |  |
| Long-lived, slow-growing sp. | -1.806 | 0.55 | -3.278 | 0.001 | * |  |  |  |  |  |  |  |  |  |
| Short-lived, fast-growing sp. | -1.05 | 0.391 | -2.705 | 0.006 | * |  |  |  |  |  |  |  |  |  |
| DBH: Long-lived, slow-growing sp. | 0.06 | 0.001 | 3.531 | 0.001 | * |  |  |  |  |  |  |  |  |  |
| DBH: Short-lived, fast-growing sp. | 0.03 | 0.001 | 3.226 | 0.001 | * |  |  |  |  |  |  |  |  |  |
|  | | | | | | | | | | | | | | |
| **Rot-holes model** | | | | | | Poisson | 0.23 | 341.1 /354.2 | -164.5 | 329.1 | 666 | 0.9 | 0.52 | 0.8 |
| Intercept | -4.251 | 0.662 | -6.416 | 1.4e-10 | * |  |  |  |  |  |  |  |  |  |
| DBH (cm) | 0.003 | 0.001 | 2.291 | 0.0220 | * |  |  |  |  |  |  |  |  |  |
| Unmanaged stand | -0.574 | 0.580 | -0.989 | 0.3226 |  |  |  |  |  |  |  |  |  |  |
| Long-lived, slow-growing sp. | 0.278 | 0.539 | 0.517 | 0.6054 |  |  |  |  |  |  |  |  |  |  |
| Short-lived, fast-growing sp. | 0.789 | 0.382 | 2.063 | 0.0391 | * |  |  |  |  |  |  |  |  |  |
|  | | | | | | | | | | | | | | |
| **Concavities model** | | | | | | Negative binomial | 0.60 | 281.6 /296.1 | -133.8 | 267.6 | 665 | 0.6638 | 0.904 | 0.856 |
| Intercept | -2.563e+00 | 1.015e+00 | -2.524 | 0.0115 | * |  |  |  |  |  |  |  |  |  |
| DBH (cm) | 4.283e-04 | 3.302e-03 | 0.130 | 0.8968 |  |  |  |  |  |  |  |  |  |  |
| Unmanaged stand | 1.669e-01 | 6.308e-01 | 0.264 | 0.7913 |  |  |  |  |  |  |  |  |  |  |
| Long-lived, slow-growing sp. | -2.046e+01 | 8.835e+03 | -0.002 | 0.9981 |  |  |  |  |  |  |  |  |  |  |
| Short-lived, fast-growing sp. | -1.938e+00 | 6.826e-01 | -2.839 | 0.0045 | * |  |  |  |  |  |  |  |  |  |
|  | | | | | | | | | | | | | | |
| **Exposed sapwood model** | | | | | | Negative binomial | 0.23 | 161.3 /163.2 | -73.7 | 147.3 | 665 | 0.826 | 0.632 | 0.968 |
| Intercept | -4.6391 | 1.5568 | -2.980 | 0.0028 | * |  |  |  |  |  |  |  |  |  |
| DBH (cm) | -0.0017 | 0.0053 | -0.331 | 0.7404 |  |  |  |  |  |  |  |  |  |  |
| Unmanaged stand | 0.7127 | 0.7145 | 0.997 | 0.3185 |  |  |  |  |  |  |  |  |  |  |
| Long-lived, slow-growing sp. | 0.2233 | 1.3627 | 0.164 | 0.8698 |  |  |  |  |  |  |  |  |  |  |
| Short-lived, fast-growing sp. | 2.2678 | 0.9472 | 2.394 | 0.0166 | * |  |  |  |  |  |  |  |  |  |
|  | | | | | | | | | | | | | | |
| **Exposed sap- and heartwood model** | | | | | | Negative binomial | 0.39 | 305.8 /312.0 | -145.9 | 291.8 | 665 | 0.269 | 0.936 | 0.96 |
| Intercept | -2.3995 | 1.1318 | -2.120 | 0.0340 | * |  |  |  |  |  |  |  |  |  |
| DBH (cm) | -0.0029 | 0.0033 | -0.882 | 0.3778 |  |  |  |  |  |  |  |  |  |  |
| Unmanaged stand | -0.9067 | 0.9144 | -0.992 | 0.3214 |  |  |  |  |  |  |  |  |  |  |
| Long-lived, slow-growing sp. | -1.0885 | 1.2225 | -0.890 | 0.3732 |  |  |  |  |  |  |  |  |  |  |
| Short-lived, fast-growing sp. | 1.7381 | 0.5895 | 2.948 | 0.0032 | * |  |  |  |  |  |  |  |  |  |
|  | | | | | | | | | | | | | | |
| **Crown deadwood model** | | | | | | Negative binomial | 0.21 | 543.1 /593.7 | -264.6 | 529.1 | 665 | 0.826 | 0.128 | 1 |
| Intercept | -4.8962 | 0.4655 | -10.518 | < 2e-16 | * |  |  |  |  |  |  |  |  |  |
| DBH (cm) | 0.0079 | 0.0012 | 6.444 | 1.16e-10 | * |  |  |  |  |  |  |  |  |  |
| Unmanaged stand | 0.1693 | 0.2420 | 0.700 | 0.4841 |  |  |  |  |  |  |  |  |  |  |
| Long-lived, slow-growing sp. | 0.8251 | 0.3371 | 2.447 | 0.0144 | * |  |  |  |  |  |  |  |  |  |
| Short-lived, fast-growing sp. | -0.0003 | 0.3185 | -0.001 | 0.9992 |  |  |  |  |  |  |  |  |  |  |
|  | | | | | | | | | | | | | | |
| **Epiphytes model** | | | | | | Negative binomial | 0.49 | 1652.6/1751.8 | -819.3 | 1638.6 | 665 | 1 | 0.984 | 0.776 |
| Intercept | -2.2332 | 0.6782 | -3.292 | 0.0009 | * |  |  |  |  |  |  |  |  |  |
| DBH (cm) | 0.0029 | 0.0010 | 2.864 | 0.0041 | * |  |  |  |  |  |  |  |  |  |
| Unmanaged stand | 0.4529 | 0.8577 | 0.528 | 0.5974 |  |  |  |  |  |  |  |  |  |  |
| Long-lived, slow-growing sp. | 0.1982 | 0.2487 | 0.797 | 0.4254 |  |  |  |  |  |  |  |  |  |  |
| Short-lived, fast-growing sp. | 1.4117 | 0.2118 | 6.665 | 2.64e-11 | * |  |  |  |  |  |  |  |  |  |
|  | | | | | | | | | | | | | | |
| **Microsoils model** | | | | | | Negative binomial | 0.17 | 375.6 / 377.6 | -181.8 | 363.6 | 666 | 0.189 | 0.696 | 1 |
| Intercept | -2.6537 | 0.5950 | -4.460 | 8.2e-06 | * |  |  |  |  |  |  |  |  |  |
| DBH (cm) | 0.0012 | 0.0017 | 0.719 | 0.4722 |  |  |  |  |  |  |  |  |  |  |
| Unmanaged stand | -0.2306 | 0.4685 | -0.492 | 0.6225 |  |  |  |  |  |  |  |  |  |  |
| Long-lived, slow-growing sp. | -1.6396 | 0.7393 | -2.218 | 0.0266 | * |  |  |  |  |  |  |  |  |  |
| Short-lived, fast-growing sp. | -0.396 | 0.361 | -1.095 | 0.2735 |  |  |  |  |  |  |  |  |  |  |
|  | | | | | | | | | | | | | | |

Appendix 4. The estimated effect of the interaction of tree DBH and species group (based on successional character and compartmentalization capacity) on mean TreM abundance and richness at tree-level. Ribbons represent the 95% confidence intervals. Only significant results are included in the graphs. Forest management was not a significant predictor in any of the models.


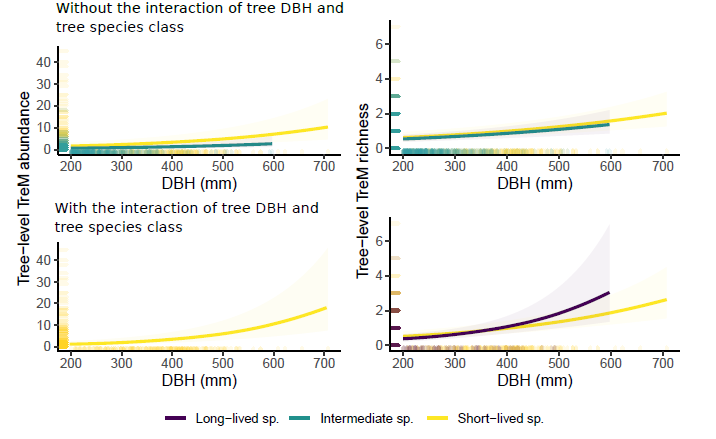

Supplement: Supplementary file 1 — Appendix S1 [file ECE3-13-e10238-s002.docx]
